# Elevated ITGA1 levels in type 2 diabetes: implications for cardiac function impairment

**DOI:** 10.1007/s00125-024-06109-4

**Published:** 2024-02-27

**Authors:** Mengqi Su, Yilin Hou, Sidong Cai, Wenpeng Li, Yinxia Wei, Run Wang, Min Wu, Mingya Liu, Junlei Chang, Kelaier Yang, Kaihang Yiu, Cong Chen

**Affiliations:** 1 Department of Cardiology, The University of Hong Kong-Shenzhen Hospital, Shenzhen, China; 2 Institute of Biomedicine and Biotechnology, Shenzhen Institute of Advanced Technology, Chinese Academy of Sciences, Shenzhen, China; 3 Department of Otorhinolaryngology, The First Affiliated Hospital of Sun Yat-sen University, Guangzhou, China; 4 Department of Cardiology, The First Affiliated Hospital of Harbin Medical University, Harbin, China; 5 Department of Endocrinology and Metabolism, Shenzhen University General Hospital, Shenzhen, China; 6 Department of Cardiology, The University of Hong Kong, Queen Mary Hospital, Hong Kong, China

**Keywords:** Cardiac remodelling, Echocardiography, Heart failure with preserved ejection fraction, HFpEF, ITGA1, Plasma integrin α1, Type 2 diabetes

## Abstract

**Aims/hypothesis:**

Type 2 diabetes mellitus is known to contribute to the development of heart failure with preserved ejection fraction (HFpEF). However, identifying HFpEF in individuals with type 2 diabetes early on is often challenging due to a limited array of biomarkers. This study aims to investigate specific biomarkers associated with the progression of HFpEF in individuals with type 2 diabetes, for the purpose of enabling early detection and more effective management strategies.

**Methods:**

Blood samples were collected from individuals with type 2 diabetes, both with and without HFpEF, for proteomic analysis. Plasma integrin α1 (ITGA1) levels were measured and compared between the two groups. Participants were further categorised based on ITGA1 levels and underwent detailed transthoracic echocardiography at baseline and during a median follow-up period of 30 months. Multivariable linear and Cox regression analyses were conducted separately to assess the associations between plasma ITGA1 levels and changes in echocardiography indicators and re-hospitalisation risk. Additionally, proteomic data for the individuals’ left ventricles, from ProteomeXchange database, were analysed to uncover mechanisms underlying the change in ITGA1 levels in HFpEF.

**Results:**

Individuals with type 2 diabetes and HFpEF showed significantly higher plasma ITGA1 levels than the individuals with type 2 diabetes without HFpEF. These elevated ITGA1 levels were associated with left ventricular remodelling and impaired diastolic function. Furthermore, during a median follow-up of 30 months, multivariable analysis revealed that elevated ITGA1 levels independently correlated with deterioration of both diastolic and systolic cardiac functions. Additionally, higher baseline plasma ITGA1 levels independently predicted re-hospitalisation risk (HR 2.331 [95% CI 1.387, 3.917], *p*=0.001). Proteomic analysis of left ventricular myocardial tissue provided insights into the impact of increased ITGA1 levels on cardiac fibrosis-related pathways and the contribution made by these changes to the development and progression of HFpEF.

**Conclusions/interpretation:**

ITGA1 serves as a biomarker for monitoring cardiac structural and functional damage, can be used to accurately diagnose the presence of HFpEF, and can be used to predict potential deterioration in cardiac structure and function as well as re-hospitalisation for individuals with type 2 diabetes. Its measurement holds promise for facilitating risk stratification and early intervention to mitigate the adverse cardiovascular effects associated with diabetes.

**Data availability:**

The proteomic data of left ventricular myocardial tissue from individuals with type 2 diabetes, encompassing both those with and without HFpEF, is available from the ProteomeXchange database at http://proteomecentral.proteomexchange.org.

**Graphical Abstract:**

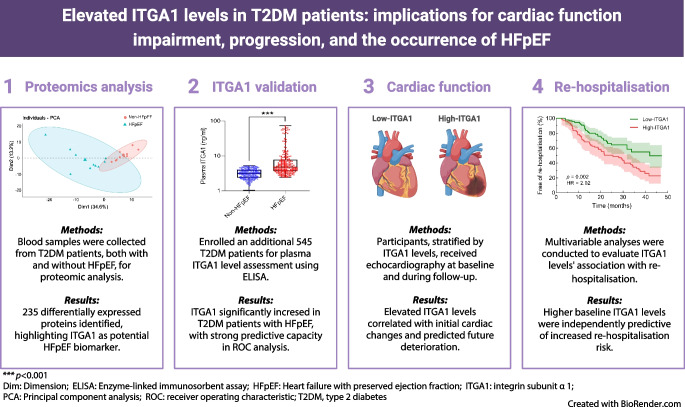

**Supplementary Information:**

The online version contains peer-reviewed but unedited supplementary material available at 10.1007/s00125-024-06109-4.


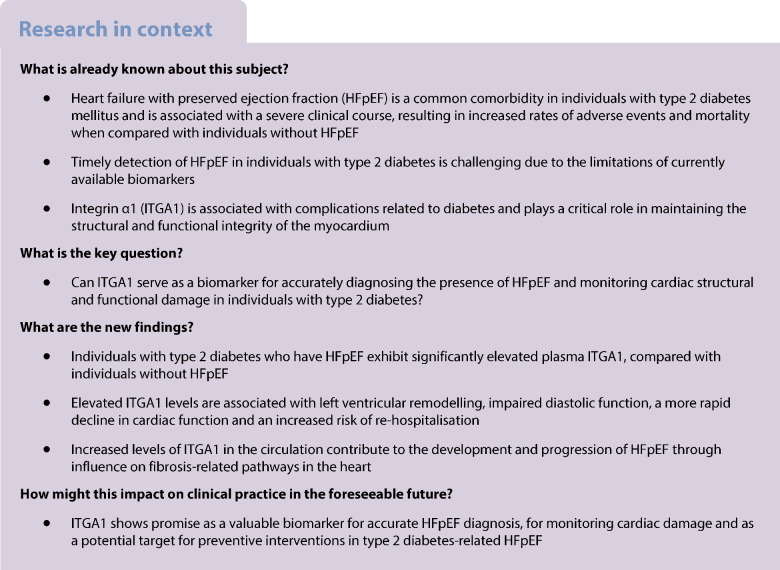


## Introduction

Heart failure is a chronic and complex disease that poses a significant public health concern. Heart failure with preserved ejection fraction (HFpEF) constitutes approximately 50–55% of heart failure and its prevalence is increasing at a rate of around 1% per year [1]. Unlike heart failure with reduced ejection fraction, effective strategies for HFpEF management remain elusive [2]. Therefore, there is a pressing need to better understand the pathophysiology of HFpEF and screen potential risk factors to improve its prevention and management.

Type 2 diabetes mellitus is a common comorbidity in HFpEF; the presence of type 2 diabetes in individuals with HFpEF is associated with a higher rate of hospitalisation and mortality when compared with the absence of diabetes [3]. Approximately 45% of individuals with HFpEF have type 2 diabetes and the occurrence of comorbid type 2 diabetes is rising particularly among those with newly diagnosed HFpEF [4]. Unfortunately, the lack of obvious symptoms and limited biomarkers contribute to the delayed detection and treatment of HFpEF in individuals with type 2 diabetes [5]. Recent advances in peripheral blood analytical techniques and proteomics analysis have provided valuable insights into potential peripheral blood biomarkers for identifying HFpEF [6–8]. However, specific biomarkers for type 2 diabetes are limited and their relationship with heart structure and function, as well as future trends, has not been thoroughly analysed for most biomarkers [9]. To offset this deficiency, we conducted a plasma proteomics study on a well-matched subset of individuals with type 2 diabetes with and without HFpEF. Our analysis revealed a significant elevation in integrin α1 (ITGA1) levels among individuals with type 2 diabetes and HFpEF when compared with individuals with type 2 diabetes without HFpEF.

ITGA1 belongs to the integrin family, which consists of 18 α and 8 β subunits [10]. Traditionally, integrins are considered to be membrane-bound proteins that regulate cell-to-cell and cell-to-extracellular-matrix interactions [11]. However, it has been recently discovered that integrin subunits can exist in soluble forms in the circulation and have potential utility as diagnostic or prognostic markers for certain diseases [12]. For example, integrin β8 has been identified as a promising serum marker for the diagnosis, prognosis and surveillance of advanced colorectal cancer [13]. Additionally, the circulating levels of integrins β1, β2 and β3 can serve as diagnostic markers for venous thromboembolism [14]. ITGA1 is involved in cardiomyocyte adhesion and collagen secretion of myocardial fibroblasts, playing a critical role in cardiac remodelling [15, 16]. However, the relationship between ITGA1 plasma levels and the progression of left ventricular remodelling remains poorly understood. Therefore, this study aimed to validate ITGA1 as a diagnostic biomarker for identifying individuals with type 2 diabetes who are at a higher risk of developing HFpEF. Additionally, we aimed to establish a correlation between ITGA1 levels and abnormal cardiac structure and function, as well as explore its potential for predicting future cardiac deterioration and re-hospitalisation.

## Methods

### Study design

**Fig. 1:**
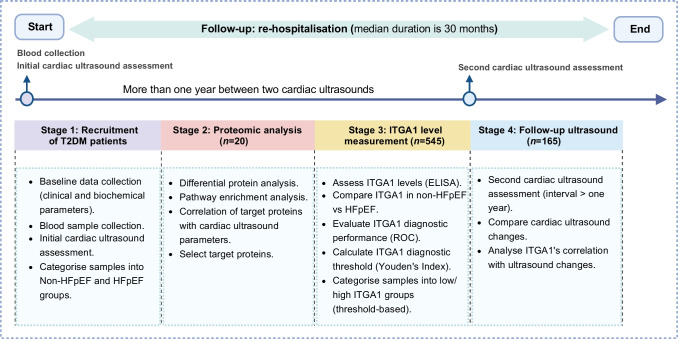
Flow diagram of the study process. We first recruited individuals with type 2 diabetes and collected baseline data. The participants were then categorised into non-HFpEF and HFpEF groups based on diagnostic criteria. In the proteomic analysis phase, we identified differentially expressed proteins related to HFpEF and correlated these proteins with echocardiographic data, finding that ITGA1 correlated significantly with cardiac structure and function. Subsequently, we focused on ITGA1, measuring its levels in both non-HFpEF and HFpEF groups and assessing its diagnostic utility. The final stage involved follow-up cardiac ultrasound assessments and monitoring re-hospitalisation rates. The follow-up period, commencing from participant recruitment and initial blood sample collection, extended over a median duration of 30 months. T2DM, type 2 diabetes

In our study on type 2 diabetes and HFpEF, we first recruited individuals with type 2 diabetes, collecting baseline data including demographics, clinical measurements and laboratory tests. These individuals were then categorised into non-HFpEF and HFpEF groups based on diagnostic criteria. In the proteomic analysis phase, we identified differentially expressed proteins related to HFpEF. Notably, the ‘hypertrophic cardiomyopathy’ pathway, which included proteins such as ITGA1, myosin heavy chain 7 (MYH7), desmin (DES), actin β (ACTB), IGF1 and calcium voltage-gated channel auxiliary subunit α2δ1 (CACNA2D1), was of particular interest due to its strong link to HFpEF. We correlated these proteins with echocardiographic data, finding ITGA1 to have significant correlations with cardiac structure and function. Subsequently, we focused on ITGA1, measuring its levels in both non-HFpEF and HFpEF groups and assessing its diagnostic utility. The final stage involved follow-up cardiac ultrasound assessments and monitoring of re-hospitalisation rates. The follow-up period, commencing from participant recruitment and initial blood sample collection, extended over a median duration of 30 months (Fig. 1).

### Study population

All participants were recruited from the Cardiovascular Medical Centers of the University of Hong Kong - Shenzhen Hospital from June 2019 to June 2021. HFpEF was diagnosed according to the European Society of Cardiology guideline [17]: (1) presence of symptoms and/or signs of heart failure; (2) left ventricular ejection fraction (LVEF) ≥50%; and (3) N-terminal pro-B-type natriuretic peptide (NT-proBNp) >125 pg/ml. Type 2 diabetes was diagnosed using ADA guidelines [18]. Individuals meeting any of the following criteria were excluded: (1) LVEF <50% at any time; (2) isolated right heart failure due to pulmonary disease; (3) dyspnoea due to non-cardiac causes such as pulmonary disease, anaemia or severe obesity; or (4) severe valvular disease, infiltrative cardiomyopathy, congenital heart disease, chronic obstructive pulmonary disease (Global Initiative for Chronic Obstructive Lung Disease [GOLD] stage 3 or 4) or pericardial disease. The study was approved by the local ethics committee and written informed consent was obtained from all participants.

### Clinical and biochemical data

Clinical measurements and blood sampling were conducted after participants had fasted overnight for at least 8 h. Age, sex (determined based on self-reported information) and detailed medical histories, including smoking status and the presence of hypertension, atrial fibrillation or coronary artery disease were recorded. Anthropometric measurements (body weight and height) were recorded and BMI was calculated. BP was measured at the end of the echocardiography examination after a 5 min rest. Fasting blood samples were collected for measuring HbA_1c_, glucose, lipid profile and serum creatinine levels and stored at −80°C for additional assays [19]. ITGA1 levels were measured using an ELISA kit (FineTest, China). The kit had an intra- and inter-assay coefficient of variation <8% and <10%, respectively, and a sensitivity limit of 0.188 ng/ml.

### Sample preparation and proteomic analysis

Serum samples were thawed, centrifuged to remove debris, and high-abundance proteins were removed. Protein concentration was determined, and the proteins were reduced, alkylated and digested using the filter-aided sample preparation (FASP) method. Peptides were recovered by centrifugation and desalted using a Strata X SPE column (Phenomenex, CA, USA). Tryptic peptides were separated on a reverse-phase column with a solvent gradient. Orbitrap Exploris 480 mass spectrometer (Thermo Fisher Scientific, MA, USA) was used for peptide analysis, adjusting scan resolutions. Abundant precursors were selected for MS/MS analysis with high-energy collision dissociation (HCD) fragmentation. Proteome Discoverer search engine (v.2.4) was used for data analysis against a human protein database with specified criteria [20]. Mass error tolerances and false discovery rate (FDR) thresholds were adjusted. The detailed steps were followed as described in previous studies [21].

To further investigate the potential mechanisms linking elevated plasma ITGA1 levels to cardiac dysfunction and the development of HFpEF, we retrieved proteomic data for left ventricular myocardial tissue from individuals with type 2 diabetes with and without HFpEF from the ProteomeXchange database (http://proteomecentral.proteomexchange.org) and conducted a comparative proteomic analysis.

### Echocardiography measurement

Standard two-dimensional echocardiography and tissue Doppler imaging was performed on recruited participants with a commercially available echocardiography system (VingmedE9; General Electric Vingmed Ultrasound, Horten, Norway) by skilled operators who were blinded to the participants’ clinical and biochemical characteristics. Participants were in the lateral decubitus position, and a 3.5 MHz transducer (General Electric Healthcare, IL, USA) was used to capture images and digitally store them in cine-loop format. Left ventricular end diastolic dimension (LVDD), left ventricular end systolic dimension (LVDS) and interventricular septal dimension at end-diastole (IVSD) were measured by the leading-edge-to-leading-edge method from two-dimensional guided M-mode tracings recorded at the parasternal long-axis view. Left ventricular mass was calculated according to the Devereux formula. Left ventricular volume and LVEF were measured using modified biplane Simpson’s method in apical four- and two-chamber views. Doppler imaging was applied to assess left ventricular diastolic function in apical four-chamber view. The transmitral early diastolic peak velocity (E) wave and transmitral late diastolic peak velocity (A) wave were measured, and the E/A ratio was calculated. The early diastolic peak velocity of mitral valve at septal or lateral annulus (e′) was measured by tissue Doppler imaging, and the average E/e′ was calculated [22].

### Statistical analysis

Continuous variables were presented as mean ± SD, as well as frequencies and percentages for categorical variables. Normal distribution was assessed through the Kolmogorov–Smirnov test. For normally distributed variables, independent samples *t* tests were used to determine differences. Non-normally distributed or heterogeneous data were analysed using non-parametric tests. Categorical variables were compared using the χ^2^ test. In the small cohorts, variables including age, sex, duration of diabetes, BMI, smoking status, comorbidities and laboratory findings (glucose, HbA_1c_, albumin, triacyglycerol, total cholesterol, HDL-cholesterol, LDL-cholesterol, uric acid, and creatinine) were matched (*p*>0.05 for difference between groups).

The predictive ability of serum ITGA1 for HFpEF was assessed using the AUC in the receiver operating characteristic (ROC) curve. ITGA1’s Youden’s index was derived from the ROC curves. Changes in clinical characteristics and echocardiography indicators between baseline and follow-up were analysed using paired *t* tests or McNemar tests, as appropriate. Multivariable linear regression was used to examine the association between serum ITGA1 levels and changes in echocardiography indicators, taking into account the matching variables in the small cohorts. Kaplan–Meier survival curves with the logrank test were used to compare re-hospitalisation rates based on ITGA1 levels. The association between ITGA1 and re-hospitalisation risk was assessed using multivariable Cox regression, with significant variables from univariate analyses or biologically relevant factors included in the multivariable regression models.

IBM SPSS 26.0 (https://spss.en.softonic.com/) was used for all statistical analyses, with a two-sided *p* value <0.05 considered statistically significant.

## Results

### Baseline characteristics of the study population

Given the complex relationship between type 2 diabetes and HFpEF, identifying at-risk individuals is crucial for early intervention. To identify biomarkers that could better distinguish individuals with type 2 diabetes at higher risk of HFpEF development, a subset of 20 well-matched participants with type 2 diabetes was initially selected for a plasma proteomics study. This subset consisted of ten individuals with HFpEF and ten individuals without the condition (non-HFpEF). Electronic supplementary material (ESM) Table 1 provides detailed information on this initial study population.

Subsequently, an additional 545 participants with type 2 diabetes were enrolled for further evaluation, including blood tests and echocardiography. These participants were divided into non-HFpEF vs HFpEF groups and low-ITGA1 vs high-ITGA1 groups. Comparing the non-HFpEF and HFpEF groups, participants in the HFpEF group were found to be older, had a higher proportion of female participants and atrial fibrillation, and had a longer duration of diabetes (Table 1). Additionally, participants with HFpEF exhibited higher levels of creatinine, NT-proBNP and ITGA1, while having lower levels of albumin, compared with participants in the non-HFpEF group. In the comparison between the low-ITGA1 and high-ITGA1 groups, participants in the high-ITGA1 group were found to be older, had longer durations of diabetes, and had a higher proportion of atrial fibrillation, as well as lower proportion of arterial hypertension (Table 1). Furthermore, they presented elevated levels of uric acid, creatinine and NT-proBNP, along with lower levels of albumin.

**Table 1:** Clinical characteristics of participants categorised by HFpEF and ITGA1

Clinical characteristic	HFpEF			ITGA1		
	No (*n*=301)	Yes (*n*=244)	*p* value	Low (*n*=317)	High (*n*=228)	*p* value
Age, years	62.0±9.9	68.8±11.1	<0.001	62.1±9.8	69.0±11.3	<0.001
Female sex, *n* (%)	96 (31.9)	101 (41.4)	0.022	105 (33.1)	92 (40.4)	0.083
Duration of diabetes, years	7.9±6.9	10.9±8.3	<0.001	7.7±6.7	11.4±8.5	<0.001
BMI, kg/m^2^	25.0±3.1	24.7±3.3	0.230	25.0±3.0	24.8±3.5	0.404
Smoking, *n* (%)	90 (29.9)	73 (29.9)	0.983	104 (32.8)	59 (25.9)	0.077
Comorbidities, *n* (%)						
Arterial hypertension	253 (84.1)	195 (79.9)	0.209	271 (85.5)	177 (77.6)	0.018
Atrial fibrillation history	12 (4.0)	56 (23.0)	<0.001	22 (6.9)	46 (20.1)	<0.001
Coronary artery disease	254 (84.4)	216 (88.5)	0.163	270 (85.2)	200 (87.7)	0.395
Laboratory findings						
Glucose, mmol/l	9.26±3.81	9.89±3.92	0.059	9.40±4.11	10.11±4.59	0.063
HbA_1c_, mmol/mol	58.36±15.28	60.09±18.36	0.058	58.43±16.74	59.69±16.95	0.390
HbA_1c_, %	7.49±1.40	7.74±1.68	0.058	7.50±1.53	7.61±1.55	0.390
Albumin, g/l	44.18±4.45	41.21±4.66	<0.001	44.10±4.47	41.12±4.65	<0.001
Triacylglycerol, mmol/l	2.39±1.71	2.03±1.43	0.010	2.28±1.72	2.15±1.41	0.340
Total cholesterol, mmol/l	3.99±1.28	3.76±1.19	0.031	3.93±1.31	3.82±1.14	0.350
HDL-cholesterol, mmol/l	1.09±0.30	1.09±0.30	0.934	1.09±0.30	1.08±0.30	0.697
LDL-cholesterol, mmol/l	2.32±1.04	2.16±1.01	0.069	2.28±1.07	2.20±0.97	0.374
Uric acid, μmol/l	343.99±103.12	360.98±120.20	0.076	342.34±104.39	364.47±119.32	0.022
Creatinine, μmol/l	77.11±17.83	149.51±76.74	<0.001	77.29±17.45	154.33±81.88	<0.001
NT-proBNP, pg/ml	58.12±47.04	813.21±779.30	<0.001	75.56±50.47	843.25±912.01	<0.001
ITGA1, ng/ml	3.21±0.97	9.65±13.57	<0.001	2.93±0.67	10.41±13.68	<0.001

Data are shown as mean ± SD or *n* (%)

Among the 545 participants with type 2 diabetes included in the study, 165 agreed to participate in follow-up and returned for echocardiography examinations over a median duration of 30 months (at least 12 months after the baseline examination). These participants were then categorised into low-ITGA1 and high-ITGA1 groups. The individuals with high-ITGA1 levels were found to be older (ESM Table 2). Additionally, compared with the low-ITGA1 group, the high-ITGA1 group had a longer duration of diabetes and displayed higher levels of creatinine and NT-proBNP, along with lower levels of albumin.

### Plasma proteomic outcomes

**Fig. 2:**
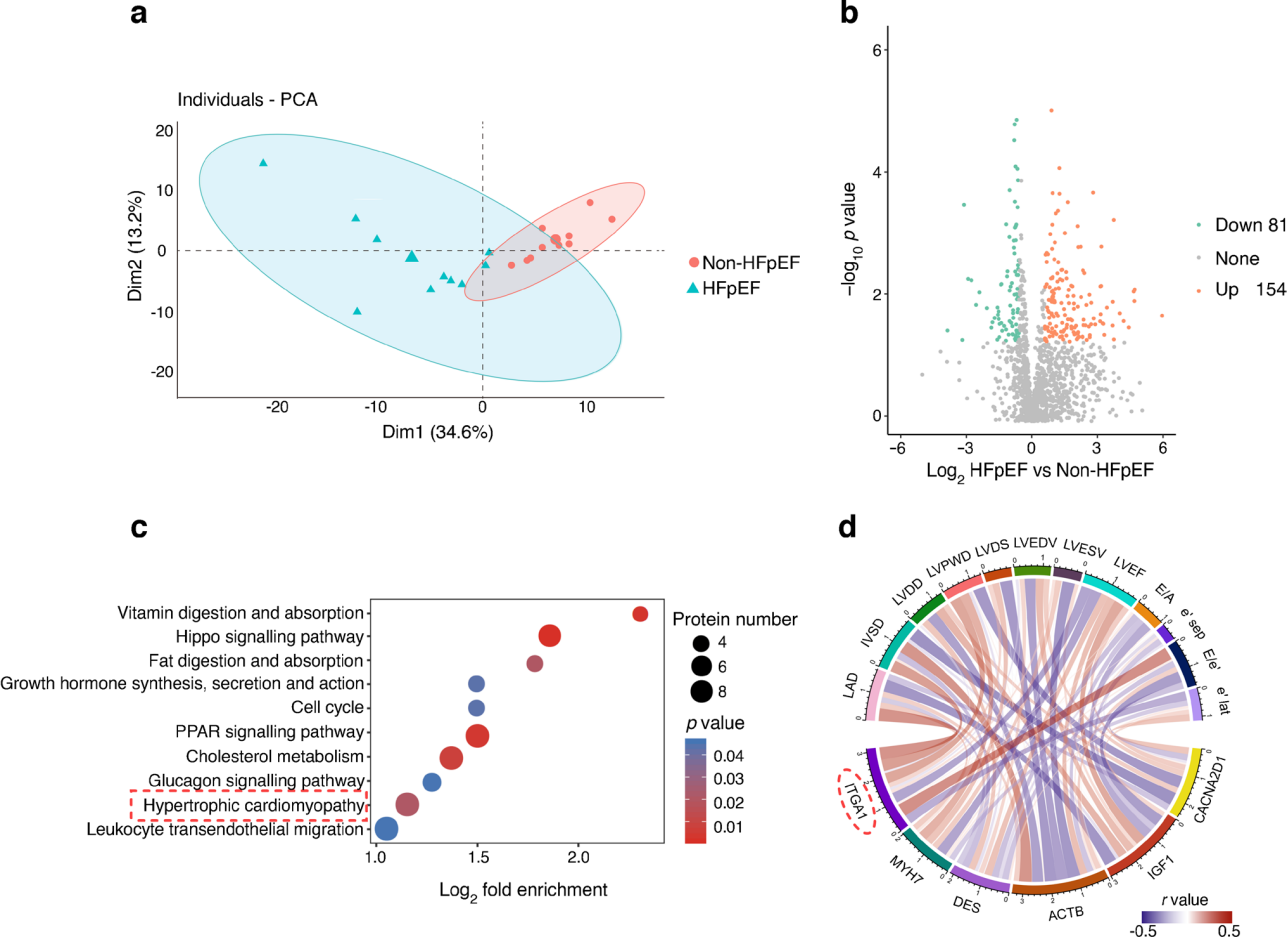
Plasma proteomic analysis comparing participants with type 2 diabetes with or without HFpEF. (**a**) Principal component analysis demonstrates distinguishable separation between the two groups. (**b**) Volcano plot showing differential proteins between the two groups. A total of 235 proteins exhibited significant alterations, including 81 downregulated and 154 upregulated proteins in the HFpEF group compared with the non-HFpEF group. (**c**) KEGG enrichment analysis revealed that the differentially expressed proteins were primarily involved in ten specific pathways. Among these pathways, ‘hypertrophic cardiomyopathy’ was closely linked to HFpEF. (**d**) Correlation analysis examining the relationship between proteins enriched in the ‘hypertrophic cardiomyopathy’ pathway and echocardiography indicators. ITGA1 demonstrated a stronger correlation with echocardiography indicators related to cardiac structure (left atrial diameter to left ventricular end systolic volume) and function (ejection fraction to e′ septal) compared with the other seven biomarkers. ACTB, actin β; CACNA2D1, calcium voltage-gated channel auxiliary subunit α2δ1; DES, desmin; Dim1, dimension 1; Dim2, dimension 2; e' lat, e' lateral; e' sep, e' septal; LAD, left atrial diameter; LVESV, left ventricular end systolic volume; MYH7, myosin heavy chain 7; PCA, principal component analysis; PPAR, peroxisome proliferator-activated receptor

The plasma proteins from participants in the non-HFpEF vs HFpEF groups were found to be distinguishable based on the results of principal component analysis (Fig. 2a). A total of 235 proteins with altered expression were identified between the two groups (81 downregulated proteins and 154 upregulated proteins in the HFpEF group compared with the non-HFpEF group) (Fig. 2b). Subsequent analysis using Kyoto Encyclopedia of Genes and Genomes (KEGG) enrichment revealed that the differentially expressed proteins were primarily involved in ten specific pathways. Among the analysed pathways, ‘hypertrophic cardiomyopathy’ stands out as a recognised contributor to HFpEF development due to its association with diastolic dysfunction and impaired heart filling (Fig. 2c). Within this pathway, ITGA1 demonstrated a stronger correlation with echocardiography indicators related to cardiac structure and function compared with the other five biomarkers (Fig. 2d and ESM Table 3). This suggests that ITGA1 could potentially serve as a valuable biomarker for assessing cardiac function in individuals with type 2 diabetes and for distinguishing those at a higher risk of developing HFpEF.

### ITGA1 levels and echocardiography indicators in individuals with type 2 diabetes with and without HFpEF

To further investigate ITGA1 as a diagnostic marker for HFpEF in individuals with type 2 diabetes, an additional cohort of 545 participants with type 2 diabetes was enrolled in the study (244 with HFpEF and 301 without HFpEF). Cardiac ultrasound assessment revealed that the participants with HFpEF exhibited several significant differences when compared with those without HFpEF, as shown in Table 2. Specifically, those with HFpEF demonstrated increased left ventricular wall thickness (*p*<0.05 for IVSD and *p*<0.01 for left ventricular posterior wall dimension [LVPWD]), mass (*p*<0.001) and volume (*p*<0.001). They also showed reduced systolic function (lower LVEF, *p*<0.001) and compromised diastolic function (lower e′ septal, e′ lateral, and E/A ratio, as well as higher average E/e′, all *p*<0.001) compared with individuals without HFpEF.

**Table 2:** Echocardiographic characteristics of participants categorised by HFpEF and ITGA1

Echocardiographic characteristic	HFpEF			ITGA1		
	No (*n*=301)	Yes (*n*=244)	*p* value	Low (*n*=317)	High (*n*=228)	*p* value
IVSD, mm	10.02±1.55	10.43±1.75	0.015	10.09±1.60	10.37±1.71	0.048
LVPWD, mm	9.85±1.61	10.33±1.98	0.003	9.93±1.61	10.25±2.02	0.038
LVDD, mm	45.95±3.90	48.39±5.86	<0.001	46.61±4.08	47.64±6.05	0.017
LVDS, mm	29.19±4.18	32.19±5.55	<0.001	29.80±4.21	31.55±5.91	<0.001
LV mass, g	159.77±41.52	185.59±58.36	<0.001	165.02±42.76	180.10±60.35	0.001
LVEDV, ml	97.94±19.14	112.89±30.13	<0.001	101.34±20.32	109.21±31.26	<0.001
LVESV, ml	33.90±10.92	43.79±18.14	<0.001	35.87±12.20	41.75±18.45	<0.001
LVEF, %	65.93±5.19	62.45±6.86	<0.001	65.11±5.98	63.35±6.46	0.001
E/A	1.11±0.26	0.93±0.56	0.002	1.04±0.29	0.89±0.56	0.013
e′ lateral, cm/s	9.07±2.57	7.66±2.41	<0.001	8.88±2.49	7.83±2.61	<0.001
e′ septal, cm/s	6.58±1.96	5.79±1.72	<0.001	6.54±2.01	5.79±1.63	<0.001
Average E/e′	10.72±3.14	14.41±6.06	<0.001	11.30±3.88	13.86±5.97	<0.001

Data are shown as mean ± SD

LV, left ventricular; LVEDV, left ventricular end diastolic volume; LVESV, left ventricular end systolic volume

**Fig. 3:**
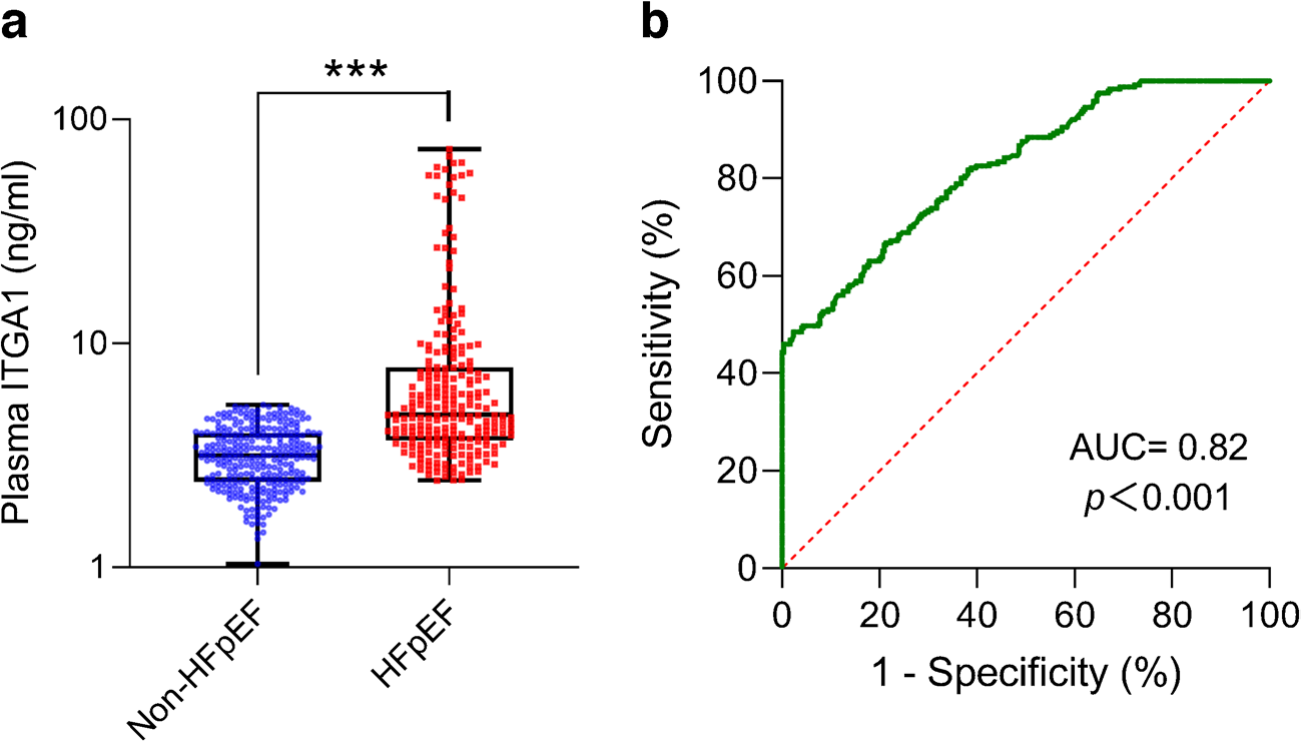
Plasma ITGA1 as a diagnostic marker for HFpEF in individuals with type 2 diabetes. (**a**) Comparison of plasma ITGA1 levels between participants with type 2 diabetes with and without HFpEF. It indicated a notable increase in individuals with type 2 diabetes who also exhibited HFpEF, compared with those without HFpEF. Box plots show median (central line), 25th and 75th percentiles (box edges), and min/max values (whiskers), ***p<0.001. (**b**) ROC curves evaluating the diagnostic ability of plasma ITGA1 for HFpEF in individuals with type 2 diabetes. The ROC analysis underscored the dependable diagnostic capability of ITGA1 for HFpEF, demonstrated by an AUC value of 0.82

The analysis of ITGA1 expression revealed its upregulation in the participants with type 2 diabetes who had HFpEF when compared with those who did not (Fig. 3a). Furthermore, ROC analysis demonstrated its strong predictive capacity for diagnosing HFpEF, as supported by an AUC value of 0.82, along with relatively higher positive (86.84%) and negative (85.49%) predictive values (Fig. 3b). Dividing the participants into low- and high-ITGA1 groups based on the Youden’s index of ITGA1, the high-ITGA1 group demonstrated increased left ventricular wall thickness (*p*<0.05 for IVSD and LVPWD), mass (*p*<0.01) and volume (*p*<0.001) (Table 2). The participants in the high-ITGA1 group also demonstrated reduced systolic function (lower LVEF, *p*<0.01) and impaired diastolic function (reduced e’ septal [*p*<0.001], e’ lateral [*p*<0.001] and E/A ratio [*p*<0.05], as well as elevated average E/e’ ratio [*p*<0.001]) compared with the low ITGA1 group.

To further explore the impact of ITGA1 on the differences in echocardiographic indices between non-HFpEF and HFpEF groups, we divided the participants into low- and high-ITGA1 groups within each HFpEF/non-HFpEF group and assessed the variations in echocardiographic indices. For the non-HFpEF participants, the high-ITGA1 group exhibited worse left ventricular diastolic function, characterised by lower e′ septal (*p*<0.05), e′ lateral (*p*<0.05) and E/A ratio (*p*<0.001) (Table 3). For participants with HFpEF, the high-ITGA1 group was associated with higher left ventricular mass (*p*<0.01), reduced systolic function (reflected by lower LVEF, *p*<0.05), as well as impaired diastolic function indicated by lower E/A ratio (*p*<0.01) and higher average E/e′ ratio (*p*<0.001). These findings suggest that high ITGA1 levels may serve as a useful predictor of cardiac diastolic dysfunction in individuals with type 2 diabetes, regardless of the presence of HFpEF. Moreover, in individuals with HFpEF, high ITGA1 levels could indicate more severe cardiac structural and functional damage.

**Table 3:** Echocardiographic variables of participants in the HFpEF and non-HFpEF groups according to the serum level of ITGA1

Echocardiographic characteristic	Non-HFpEF			HFpEF		
	Low ITGA1 (*n*=234)	High ITGA1 (*n*=67)	*p* value	Low ITGA1 (*n*=83)	High ITGA1 (*n*=161)	*p* value
IVSD, mm	9.93±1.81	9.94±1.53	0.193	10.29±1.82	10.48±1.74	0.390
LVPWD, mm	9.84±1.68	10.04±1.30	0.333	10.02±1.49	10.36±2.15	0.154
LVDD, mm	46.08±4.93	45.88±4.30	0.680	47.37±4.61	49.70±7.14	0.001
LVDS, mm	29.03±3.63	29.59±5.04	0.348	31.32±4.60	33.99±7.28	<0.001
LV mass, g	160.42±40.89	157.48±43.86	0.610	172.64±48.60	193.96±57.36	0.004
LVEDV, ml	98.45±18.27	96.52±22.08	0.471	105.66±23.19	121.68±28.20	<0.001
LVESV, ml	33.46±9.82	34.79±14.47	0.433	40.21±14.45	50.77±25.92	<0.001
LVEF, %	66.18±4.91	65.93±5.27	0.688	62.52±7.41	60.16±8.99	0.018
E/A	0.94±0.28	0.82±0.21	<0.001	0.89±0.63	0.72±0.31	0.004
e′ lateral, cm/s	6.80±1.98	6.17±1.98	0.012	5.94±1.53	5.65±1.60	0.175
e′ septal, cm/s	9.19±2.50	8.48±2.91	0.033	7.84±2.42	7.38±2.34	0.117
Average E/e′	10.27±3.03	10.98±3.39	0.101	13.20±5.07	15.93±7.25	<0.001

Data are shown as mean ± SD

LV, left ventricular; LVEDV, left ventricular end diastolic volume; LVESV, left ventricular end systolic volume

### Association between ITGA1 levels and changes in echocardiography indicators

Among the 165 participants who underwent follow-up echocardiography examinations, 90 had low ITGA1 levels and 75 had high ITGA1 levels. Comparing the follow-up assessments with the baseline measurements, participants in the low-ITGA1 group did not show significant differences in most echocardiography indices, except for a minor decrease in the E/A ratio (Table 4 and ESM Fig. 1). In contrast, participants in the high-ITGA1 group exhibited noteworthy changes in several echocardiography indicators. Specifically, the high-ITGA1 group showed a significant increase in left ventricular wall thickness (*p*<0.01 for LVPWD), mass (*p*<0.001) and LV volume (*p*<0.01 for left ventricular end diastolic volume [LVEDV] and *p*=0.01 for left ventricular end systolic volume [LVESV]) during the follow-up period. Additionally, the high-ITGA1 group displayed reduced systolic function, as indicated by a decreased LVEF (*p*<0.01), and compromised diastolic function, characterised by reduced e′ septal (*p*<0.05), e′ lateral (*p*<0.05) and E/A ratio (*p*<0.05), as well as increased average E/e′ (*p*<0.05) at follow-up.

**Table 4:** Changes in echocardiographic measures from baseline to follow-up

Echocardiographic characteristic	Low ITGA1 (*n*=90)			High ITGA1 (*n*=75)		
	Baseline	Follow-up	*p* value	Baseline	Follow-up	*p* value
IVSD, mm	9.93±1.81	9.94±1.53	0.956	10.09±1.77	10.46±1.95	0.102
LVPWD, mm	9.65±1.47	10.01±2.90	0.259	9.50±1.35	10.17±1.56	0.004
LVDD, mm	46.65±4.93	46.68±4.03	0.956	47.72±5.53	50.03±7.51	0.015
LVDS, mm	30.31±3.96	30.61±6.01	0.638	30.85±6.07	34.37±8.64	0.004
LV mass, g	179.45±56.54	182.44±67.23	0.134	182.39±64.24	199.60±62.19	<0.001
LVEDV, ml	101.81±21.39	102.53±26.38	0.775	107.48±31.08	121.22±24.15	0.008
LVESV, ml	35.42±15.81	39.37±19.26	0.087	41.44±22.14	52.05±21.85	0.010
LVEF, %	64.71±6.10	62.57±9.23	0.051	63.31±7.63	59.50±8.72	0.007
E/A	1.04±0.23	0.87±0.21	0.034	0.92±0.12	0.78±0.20	0.012
e' lateral, cm/s	6.47±2.20	6.62±2.15	0.574	6.00±1.60	5.58±1.47	0.040
e′ septal, cm/s	8.50±2.82	8.36±2.61	0.657	7.88±3.08	7.08±2.83	0.045
Average E/e′	10.62±3.87	10.76±4.70	0.767	12.09±3.74	14.24±3.30	0.015

Data are shown as mean ± SD

LV, left ventricular; LVEDV, left ventricular end diastolic volume; LVESV, left ventricular end systolic volume

To further investigate the relationship between plasma ITGA1 levels and changes in echocardiography indicators, linear regression analysis was conducted. In univariate linear regression, high ITGA1 levels at baseline were significantly associated with a decrease in LVEF and E/A ratio, and an increase in E/e′ ratio (all with *p*<0.05) (ESM Table 4). In the multivariable linear regression model, even after adjusting for baseline echocardiography indicators, sex, smoking status, duration of diabetes, total cholesterol, LDL-cholesterol and NT-proBNP, high baseline levels of ITGA1 remained independently associated with a decrease in LVEF and an increase in both the E/A ratio (*p*=0.001) and the E/e′ ratio (*p*=0.001) (Table 5). This suggests that an elevated circulating level of ITGA1 is an independent predictor of heart dysfunction, encompassing impaired diastolic and systolic functions, in type 2 diabetes patients.

**Table 5:** Multiple linear regression showing the association between change in LVEF, E/A and average E/e′, and plasma ITGA1 levels

Variable	△LVEF (%)		△E/A		△E/e′	
	Standardised β	*p* value	Standardised β	*p* value	Standardised β	*p* value
Baseline cardiac variables	−0.40	0.001	−0.46	0.001	−0.32	0.001
ITGA1	−0.26	0.007	−0.15	0.030	0.29	0.002
Sex	−0.17	0.056	0.01	0.116	−0.19	0.032
Smoking	0.17	0.057	−0.20	0.840	−0.04	0.605
Duration of diabetes, years	0.01	0.917	−0.01	0.991	−0.01	0.975
Total cholesterol, mmol/l	0.07	0.509	0.06	0.732	0.02	0.943
LDL-cholesterol, mmol/l	0.34	0.072	0.28	0.779	−0.03	0.875
NT-proBNP, pg/ml	−0.23	0.005	−4.04	0.001	−0.02	0.883

Baseline cardiac variables indicate baseline LVEF (for change in LVEF), baseline E/A (for change in E/A) and baseline average E/e′ (for change in average E/e′), respectively

### Association between ITGA1 levels and re-hospitalisation

**Fig. 4:**
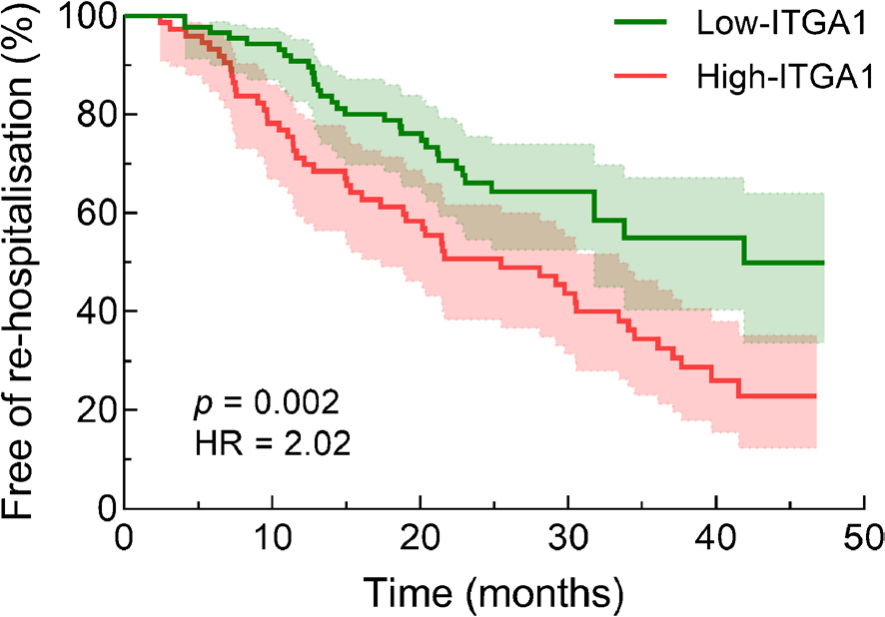
Kaplan–Meier survival curve for re-hospitalisation according to plasma ITGA1 levels. Participants in the high-ITGA1 group (*n*=75) had a significantly increased risk of re-hospitalisation compared with those in the low-ITGA1 group (*n*=90)

During a median duration of 30 months, re-hospitalisation occurred in 33 participants (36.7%) in the low-ITGA1 group and in 51 participants (68.0%) in the high-ITGA1 group. The Kaplan–Meier survival curve illustrated that those participants in the high-ITGA1 group had a significantly increased risk of re-hospitalisation compared with those in the low ITGA1 group (Fig. 4, *p*<0.01). Moreover, a multivariate Cox regression analysis revealed that the baseline ITGA1 level was independently associated with re-hospitalisation (HR 2.331 [95% CI 1.387, 3.917], *p*=0.001) after adjusting for age, duration of diabetes, smoking, atrial fibrillation history and baseline creatinine levels (Table 6). This indicates that a higher plasma ITGA1 level is indicative of a significantly elevated risk of re-hospitalisation.

**Table 6:** Cox regression analysis for re-hospitalisation in participants with type 2 diabetes

Clinical characteristic	Univariate analysis			Multivariate analysis		
	HR	95% CI	*p* value	HR	95% CI	*p* value
Age	1.020	1.002, 1.039	0.031	1.003	0.981, 1.025	0.814
Sex (male vs female)	0.725	0.466, 1.127	0.153			
Duration of diabetes	1.034	1.008, 1.061	0.009	1.024	0.994, 1.055	0.123
BMI	1.005	0.963, 1.049	0.807			
Smoking (yes vs no)	1.824	1.055, 3.154	0.031	1.561	0.851, 2.863	0.150
Arterial hypertension (yes vs no)	1.541	0.890, 2.669	0.122			
Atrial fibrillation history (yes vs no)	1.907	1.116, 3.258	0.018	1.584	0.878, 2.860	0.127
Coronary artery disease (yes vs no)	0.790	0.344, 1.816	0.579			
Glucose	0.989	0.952, 1.027	0.568			
HbA_1c_	1.049	0.970, 1.134	0.233			
Albumin	0.992	0.977, 1.008	0.323			
Triacylglycerol	0.996	0.864, 1.148	0.956			
Total cholesterol	0.885	0.771, 1.016	0.082			
HDL-cholesterol	0.841	0.483, 1.467	0.543			
LDL-cholesterol	1.010	0.975, 1.052	0.405			
Uric acid	1.000	0.999, 1.002	0.736			
Creatinine	1.001	1.000, 1.002	0.026	1.001	0.999, 1.002	0.349
NT-proBNP	1.000	0.999, 1.001	0.509			
ITGA1 level (high vs low)	2.017	1.299, 3.131	0.002	2.331	1.387, 3.917	0.001

### Mechanisms of elevated ITGA1 levels in HFpEF

Circulating proteins play a crucial role in heart biology, and changes in their levels contribute to various CVDs, such as heart failure, hypertension and ischaemic heart disease [23, 24]. To further investigate the potential mechanisms linking elevated plasma ITGA1 levels to cardiac dysfunction and the development of HFpEF, we retrieved proteomic data for left ventricular myocardial tissue from individuals with type 2 diabetes with and without HFpEF from the ProteomeXchange database (http://proteomecentral.proteomexchange.org, accessed on 10 July 2023) and conducted a comparative proteomic analysis. We observed significant differences in protein expression between the two groups (ESM Fig. 2a). Subsequent analysis using a protein–protein interaction (PPI) network identified 15 proteins as key targets of ITGA1 among the differentially expressed proteins, including seven that were downregulated and eight that were upregulated, in the HFpEF group (ESM Fig. 2b, c; ESM Table 5). Both Gene Ontology (GO) and KEGG enrichment analyses revealed that these proteins were mainly associated with myocardial fibrosis-related pathways (ESM Fig. 2d, e). These findings suggest that elevated ITGA1 levels may play a role in the promotion of myocardial fibrosis, thereby contributing to the development of HFpEF in individuals with type 2 diabetes.

## Discussion

Type 2 diabetes is a known trigger for HFpEF, affecting ventricular relaxation/stiffness and coronary microvascular function. Individuals with type 2 diabetes who have HFpEF often experience a severe clinical course, resulting in increased rates of adverse events and mortality compared with individuals who do not have HFpEF [25]. However, the timely detection of HFpEF in individuals with type 2 diabetes is often overlooked due to the limitations of currently available biomarkers. To address this challenge, we conducted proteomics analysis using blood samples collected from individuals with type 2 diabetes with and without HFpEF. The analysis revealed significantly higher levels of plasma ITGA1 in those with HFpEF compared with those without HFpEF. Moreover, the participants with elevated ITGA1 levels demonstrated left ventricular remodelling, impaired diastolic function and a more rapid decline in cardiac function, along with an increased risk of re-hospitalisation over the follow-up period of 30 months.

ITGA1 is a member of the integrin family, which consist of α and β receptor subunits and act as transmembrane cell-adhesion molecules [10]. Integrins, including ITGA1, play a critical role in maintaining the structural and functional integrity of the myocardium in a healthy heart. However, in the presence of heart disease, integrin expression and function can be altered in response to abnormal stress signals, leading to cardiac remodelling [26, 27]. Suppressing ITGA1 expression has been shown to alleviate aggregation in the pathogenesis of cardiomyopathies, highlighting the strong association between abnormal ITGA1 expression and cardiac dysfunction [16]. Integrins, in general, have also been associated with cardiac dysfunction in diabetes. For instance, in diabetic cardiomyopathy, increased levels of integrin α11 expression can stimulate and activate TGF-β2, leading to collagen synthesis and myofibroblast differentiation and contributing to the development of fibrotic tissue [28]. Integrin α5 has been implicated in the regulation of vascular complications in type 1 diabetes [29]. In mouse models of diabetes, the combination of sKL and integrin β1 triggers the activation of the ERK1/2 pathway, leading to selective insulin resistance and myocardial fibrosis. Furthermore, ITGA1 has been implicated in diabetes-related complications. In mice with high-fat-induced insulin resistance, increased ITGA1 expression in hepatocytes is associated with impaired hepatic glucose metabolism, while the deletion of ITGA1 improves fatty liver conditions [30]. These findings suggest the potential involvement of ITGA1 in the cardiac remodelling associated with diabetes.

While integrin is primarily known as a transmembrane receptor, previous studies have demonstrated the existence of soluble forms of integrin in the bloodstream. These soluble forms are generated through proteolytic cleavage of the extracellular domain of integrin and have emerged as potential biomarkers for various diseases [31, 32]. For instance, high levels of circulating integrin have been implicated as diagnostic markers for venous thromboembolism [14] and enrichment of integrin αvβ1 has been observed in the circulation of individuals with advanced stages of breast cancer [33]. In individuals with colorectal cancer, the serum concentration of ITGA1 was also found to be significantly higher compared with that in healthy individuals and showed a significant association with metastatic (tumour, node, metastasis [TNM]) stage [34]. However, there is currently no reported research on the relationship between serum levels of ITGA1 and cardiac remodelling associated with type 2 diabetes.

In our study, we investigated the potential of ITGA1 as a diagnostic marker for HFpEF in individuals with type 2 diabetes. Additionally, we compared the echocardiographic profiles of participants categorised by ITGA1 levels at baseline and follow-up. Echocardiography, a widely accessible imaging technique, enables the identification of adverse left ventricular remodelling and diastolic dysfunction in individuals with type 2 diabetes [35]. Prior cross-sectional studies using echocardiography have highlighted various factors contributing to left ventricular hypertrophy and diastolic dysfunction in individuals with type 2 diabetes, including oxidative stress [36], autonomic dysfunction [37], microvascular disease [38], obesity [39] and poor glycaemic control [40]. Prospective echocardiography studies have also shown that longitudinal changes in left ventricular remodelling and myocardial dysfunction in individuals with type 2 diabetes are associated with factors such as retinopathy [41], B-type natriuretic peptide (BNP) [42], obesity [43] and female sex [44].

Our baseline assessments revealed that non-HFpEF participants with elevated ITGA1 levels exhibited marked left ventricular diastolic dysfunction, while in participants with HFpEF, higher ITGA1 levels were associated with increased left ventricular mass and deteriorations in both systolic and diastolic functions. This suggests that elevated ITGA1 levels could serve as an early indicator of cardiac dysfunction in individuals with type 2 diabetes without HFpEF, despite absence of overt heart failure symptoms. In contrast, in individuals with type 2 diabetes who display HFpEF, high ITGA1 levels indicate a more severe cardiac condition, encompassing both systolic and diastolic impairments, which may lead to a greater risk of adverse cardiovascular events and necessitate more intensive management. Additionally, our longitudinal analysis highlighted a significant correlation between baseline ITGA1 levels and the progression of cardiac dysfunction in individuals with type 2 diabetes. This progression, observed over the follow-up period, manifested as a decline in both diastolic and systolic functions. Furthermore, we observed a notable link between higher ITGA1 levels at baseline and an increased likelihood of re-hospitalisation during follow-up. This finding emphasises the strong relationship between ITGA1 levels and the risk of adverse clinical outcomes in these individuals.

While BNP and NT-proBNP are commonly used biomarkers for HFpEF, they primarily indicate significant cardiac damage or functional decline, reflecting notable pathological processes in the heart [45, 46]. In contrast, ITGA1 shows promise in assessing early-stage cardiac diastolic dysfunction. Our study revealed that even in individuals without HFpEF and with normal NT-proBNP levels, elevated levels of ITGA1 were consistently associated with reduced cardiac diastolic function compared with lower ITGA1 levels. These findings underscore the potential of ITGA1 as a marker for detecting early cardiac diastolic dysfunction and providing valuable insights into subclinical pathological changes that may precede the development of overt cardiac damage or functional impairment.

However, the underlying pathogenic mechanism linking elevated circulating levels of ITGA1 to adverse left ventricular remodelling and functional impairment in individuals with type 2 diabetes remains unclear. To investigate this, we utilised proteomic data from the left ventricle of individuals with type 2 diabetes with or without HFpEF. Intriguingly, we found that the proteins displaying strong associations with ITGA1 were significantly enriched in pathways related to myocardial fibrosis. Myocardial fibrosis plays a critical role in the development of HFpEF by contributing to cardiac dysfunction [47]. This process involves the excessive deposition of collagen and other extracellular matrix components in the myocardium, leading to increased myocardial stiffness and impaired relaxation during diastole. Furthermore, such fibrotic remodelling disrupts the normal architecture of the myocardium, compromising coordinated contraction and further exacerbating cardiac dysfunction [48]. Thus, our findings provide valuable insights into the molecular mechanisms by which ITGA1 influences myocardial fibrosis and contributes to the pathogenesis of HFpEF in individuals with type 2 diabetes.

### Clinical implications

The study findings have important clinical implications for people with type 2 diabetes. ITGA1 may serve as a valuable biomarker for monitoring cardiac damage, diagnosing HFpEF accurately, predicting further deterioration in cardiac structure and function, and identifying individuals at higher risk of re-hospitalisation. Integrating ITGA1 into routine diagnostic protocols could improve the identification and management of HFpEF in individuals with type 2 diabetes. Additionally, ITGA1’s role in the pathophysiology of HFpEF highlights potential avenues for targeted therapeutic interventions. Modulating the expression or function of ITGA1, through the development of new drugs or repurposing existing medications, may help prevent or mitigate the onset of HFpEF in individuals with type 2 diabetes. These targeted interventions could potentially slow disease progression, enhance cardiac function and improve overall clinical outcomes.

### Limitations

Our study had limitations, including a small sample size and a single-centre design. These may have affected the statistical power and introduced biases. Larger studies involving multiple centres are needed to confirm and extend our findings. Additionally, depending solely on proteomic data and bioinformatics analysis to demonstrate the impact of ITGA1 on HFpEF through influence on fibrotic pathways may introduce potential inaccuracies. Incorporating other approaches such as animal models or in vitro studies would provide a more comprehensive understanding of the underlying mechanisms. Future research should address these limitations to enhance the diagnostic and predictive value of ITGA1 for HFpEF.

### Conclusion

Our study provides evidence supporting the involvement of ITGA1 in HFpEF among individuals with type 2 diabetes. We found that ITGA1 levels were significantly elevated in individuals with type 2 diabetes with HFpEF. Individuals with elevated ITGA1 levels exhibited left ventricular remodelling, impaired diastolic function and a faster decline in cardiac function, correlating with an increased risk of re-hospitalisation during the follow-up period. Further investigations using proteomic data from the left ventricle revealed that increased circulating levels of ITGA1 contribute to the development and progression of HFpEF by influencing fibrosis-related pathways in the heart. Based on these findings, ITGA1 holds potential as a prognostic marker for identifying high-risk individuals with type 2 diabetes who are prone to cardiovascular complications. Its measurement could facilitate risk stratification and early intervention to mitigate the adverse effects of HFpEF.

### Supplementary Information

Below is the link to the electronic supplementary material.

Supplementary file1 (PDF 765 KB)

## Data Availability

The proteomic data of left ventricular myocardial tissue from individuals with type 2 diabetes, encompassing both those with and without HFpEF, is available from the ProteomeXchange database at http://proteomecentral.proteomexchange.org. For individual participant data related to this study, availability will commence 12 to 36 months after publication. Researchers interested in these data are invited to submit their research proposals to the corresponding author, Cong Chen, at chenc6@hku-szh.org.

## Abbreviations

A: Transmitral late diastolic peak velocity
BNP: B-type natriuretic peptide
E: Transmitral early diastolic peak velocity
e′: Early diastolic peak velocity of mitral valve at septal or lateral annulus
HFpEF: Heart failure with preserved ejection fraction
ITGA1: Integrin α1
IVSD: Interventricular septal dimension at end-diastole
KEGG: Kyoto Encyclopedia of Genes and Genomes
LVDD: Left ventricular end diastolic dimension
LVDS: Left ventricular end systolic dimension
LVEF: Left ventricular ejection fraction
LVPWD: Left ventricular posterior wall dimension
NT-proBNP: N-terminal pro-B-type natriuretic peptide
ROC: Receiver operating characteristic
