# Supplementary material for: Elevated ITGA1 levels in type 2 diabetes: implications for cardiac function impairment
Source: Diabetologia. 2024 Feb 27;67(5):850–63. doi: 10.1007/s00125-024-06109-4 (PMC10954979; doi:10.1007/s00125-024-06109-4)
Supplement: Supplementary file 1 — Supplementary file1 (PDF 765 KB) [file 125_2024_6109_MOESM1_ESM.pdf]

Supplementary information

ESM Table 1. Clinical characteristics of subjects enrolled in plasma proteomics study

Clinical characteristics	Non-HFpEF (n=10)	HFpEF (n=10)	*p*-value	Cohen's d
Age (years)	66.30±10.63	72.00±7.22	0.178	0.626
Female sex, n (%)	5 (50.0)	5 (50.0)	1.000	
Duration of diabetes (years)	9.70±8.07	9.80±9.53	0.980	0.011
BMI (kg/m^2)	24.74±3.09	25.54±3.53	0.597	0.242
Smoking, n (%)	2 (20.0)	2 (20.0)	1.000	
Comorbidities, n (%)				
Arterial hypertension	8 (80.0)	8 (80.0)	1.000	
Atrial fibrillation history	1 (10.0)	3 (30.0)	0.264	
Coronary artery disease	9 (90.0)	9 (90.0)	1.000	
Laboratory findings				
Glucose (mmol/l)	9.02±2.02	10.71±2.47	0.111	0.753
HbA1c (mmol/mol)	75.96±17.35	67.98±18.91	0.304	-0.474
HbA1c (%)	9.10±1.59	8.37±1.72	0.304	-0.474
Albumin (g/l)	41.67±1.41	40.08±2.01	0.057	-0.809
TG (mmol/l)	1.87±0.66	1.30±0.62	0.065	-0.791
TC (mmol/l)	4.08±1.36	3.43±1.30	0.283	-0.487
HDL-C (mmol/l)	1.12±0.20	1.06±0.22	0.490	-0.285
LDL-C (mmol/l)	2.62±1.24	1.97±1.09	0.229	-0.558
Uric acid (μmol/l)	309.34±69.33	288.87±128.87	0.664	-0.203
Creatinine (μmol/l)	69.00±9.61	79.60±14.18	0.066	0.805
NT-proBNP (pg/ml)	76.01±34.80	3349.40±1525.98	< 0.001	3.764

Data are presented as mean ± SD or n (%)

Abbreviations: BMI, body mass index; HbA1c, glycated hemoglobin; HDL-C, high-density lipoprotein cholesterol; ITGA1, integrin α1; LDL-C, low-density lipoprotein cholesterol; TC, total cholesterol; TG, triglycerides.

ESM Table 2. Clinical characteristics of subjects enrolled in follow-up

Clinical characteristics	Low - ITGA1 (n=90)	High - ITGA1 (n=75)	*p*-value
Age (years)	62.62±12.18	68.77±11.98	< 0.001
Female sex, n (%)	25 (27.8)	31 (41.3)	0.067
Duration of diabetes (years)	7.49±7.01	10.81±8.67	0.007
BMI (kg/m^2)	24.48±3.94	24.32±5.01	0.826
Smoking, n (%)	31 (34.4)	16 (21.3)	0.063
Comorbidities, n (%)			
Arterial hypertension	58 (64.4)	64 (85.3)	0.002
Atrial fibrillation history	10 (11.1)	11 (14.7)	0.495
Coronary artery disease	86 (95.6)	67 (89.3)	0.125
Laboratory findings			
Glucose (mmol/l)	9.49±3.7	10.1±4.83	0.862
HbA1c (mmol/mol)	55.19±23.62	54.74±28.23	0.816
HbA1c (%)	7.03±2.59	6.92±3.07	0.816
Albumin (g/l)	40.53±11.52	35.85±12.22	0.012
TG (mmol/l)	2.24±2.19	1.93±1.36	0.276
TC (mmol/l)	3.52±1.45	3.47±1.53	0.845
HDL-C (mmol/l)	0.98±0.37	0.94±0.39	0.421
LDL-C (mmol/l)	2.01±1.05	2.05±1.13	0.830
Uric acid (μmol/l)	326.36±142.14	367.86±180.54	0.100
Creatinine (μmol/l)	84.52±66.42	104.18±76.76	< 0.001
NT-proBNP (pg/ml)	560.21±254.08	1007.84±331.33	< 0.001
ITGA1 (ng/ml)	3.26±0.95	13.13±14.61	< 0.001

Data are presented as mean ± SD or n (%)

Abbreviations: BMI, body mass index; HbA1c, glycated hemoglobin; HDL-C, high-density lipoprotein cholesterol; ITGA1, integrin α1; LDL-C, low-density lipoprotein cholesterol; TC, total cholesterol; TG, triglycerides.

ESM Table 3. Correlations between 6 proteins enriched in the hypertrophic cardiomyopathy pathway and cardiac ultrasound parameters

	ITGA1		MYH7		DES		ACTB		IGF1		DACNA2D1	
	r	*p*	*r*	*p*	*r*	*p*	*r*	*p*	*r*	*p*	*r*	*p*
LAD	0.48	0.040	0.23	0.367	-0.21	0.194	0.21	0.304	-0.47	0.039	-0.24	0.129
IVSD	0.44	0.039	0.09	0.695	-0.27	0.280	0.47	0.03	-0.13	0.611	-0.49	0.040
LVDD	-0.08	0.246	0.048	0.609	0.26	0.089	-0.41	0.048	0.34	0.17	0.22	0.281
LVDS	0.18	0.484	0.27	0.284	-0.04	0.888	-0.45	0.040	0.02	0.951	0.01	0.966
LVPWD	0.42	0.028	0.35	0.050	-0.12	0.327	0.05	0.831	-0.02	0.926	-0.46	0.037
LVEDV	-0.10	0.679	0.04	0.585	0.18	0.268	-0.42	0.035	0.30	0.224	0.22	0.186
LVESV	0.12	0.329	0.23	0.368	-0.03	0.890	-0.45	0.046	0.15	0.552	-0.01	0.982
EF	-0.40	0.012	-0.39	0.045	0.27	0.270	0.29	0.139	0.39	0.111	0.22	0.272
E/A	-0.35	0.045	0.08	0.752	-0.26	0.090	-0.24	0.333	-0.36	0.123	0.06	0.817
E/e’	0.53	0.018	0.07	0.356	-0.24	0.131	0.20	0.221	-0.43	0.042	-0.25	0.310
e’ lat	0.04	0.387	0.01	0.795	-0.14	0.580	0.09	0.708	-0.24	0.147	-0.10	0.396
e’ sep	-0.35	0.053	-0.21	0.391	0.09	0.724	0.06	0.824	0.17	0.289	0.26	0.207

Abbreviations: A, trans-mitral late diastolic peak velocity; E, trans-mitral early diastolic peak velocity; e′, early diastolic peak velocity of mitral valve at septal or lateral annulus; IVSD, inter-ventricular septal dimension at end-diastole; LAD, left atrial diameter, LVDD, left ventricular end diastolic dimension; LVDS, left ventricular end systolic dimension; LVEDV, left ventricular end diastolic volume; LVESV, left ventricular end systolic volume; LVEF, LV ejection fraction.

ESM Table 4. Univariate linear regression showing the variables associated with changes in echocardiography parameters

	△LV mass (g)		△LVEF (%)		△E/A		△Average E/e’	
	β	*p*-value	β	*p*-value	β	*p*-value	β	*p*-value
Baseline cardiac parameters	-0.32	**0.001**	-0.31	**0.001**	-0.46	**0.001**	-0.13	**0.036**
ITGA1	6.22	0.374	-2.09	**0.020**	-0.13	**0.009**	1.22	**0.009**
Age (years)	0.22	0.428	0.02	0.499	0.01	0.699	-0.01	0.928
Sex	2.43	0.733	-0.95	0.299	-0.10	**0.041**	-0.85	0.076
Duration of diabetes (years)	0.95	**0.023**	-0.03	0.634	0.01	0.937	0.01	0.876
BMI (kg/m^2)	0.04	0.966	0.01	0.915	-0.01	0.919	-0.03	0.588
Smoking, n (%)	0.66	0.775	0.17	**0.043**	0.01	0.898	-0.03	0.433
Arterial hypertension	3.50	0.381	-0.79	0.123	0.02	0.438	-0.37	0.169
Atrial fibrillation history	2.28	0.857	0.29	0.857	-0.08	0.363	-0.16	0.856
Coronary artery disease	-12.93	0.306	-0.29	0.857	0.03	0.728	0.94	0.269
Glucose (mmol/l)	0.80	0.221	0.02	0.867	0.01	0.606	0.01	0.811
HbA1c (mmol/mol)	-0.11	0.930	0.08	0.613	0.02	0.099	-0.01	0.946
Albumin (g/l)	-0.14	0.660	0.06	0.119	0.01	0.671	-0.04	0.071
TG (mmol/l)	0.55	0.754	0.05	0.812	0.01	0.686	-0.02	0.880
TC (mmol/l)	-0.92	0.702	0.53	0.084	0.04	**0.027**	-0.05	0.761
HDL-C (mmol/l)	-7.99	0.392	1.56	0.194	0.11	0.084	-0.16	0.799
LDL-C (mmol/l)	-2.01	0.545	0.87	**0.041**	0.05	**0.036**	-0.08	0.735
Uric acid (μmol/l)	-0.01	0.959	0.01	0.318	0.01	0.051	0.01	0.857
Creatinine (μmol/l)	0.02	0.362	-0.01	0.267	0.01	0.099	0.01	0.200
NT-proBNP (pg/ml)	0.01	0.195	-0.01	**0.013**	-0.01	**0.001**	-0.01	0.740

Baseline cardiac parameters indicate baseline LV mass (for change in LV mass), baseline LVEF (for change in LVEF), baseline E/A (for change in E/A), and baseline average E/e’ (for change in average E/e’), respectively.

Abbreviations: BMI, body mass index; HbA1c, glycated hemoglobin; HDL-C, high-density lipoprotein cholesterol; ITGA1, integrin α1; LDL-C, low-density lipoprotein cholesterol; TC, total cholesterol; TG, triglycerides.

ESM Table 5. List of significant differentially expressed proteins exhibiting a key relationship with ITGA1

Protein name	Gene	Log2FC	Expression	*p*-value
Actin, cytoplasmic 2	*ACTG*	3.03	Up	0.003
Myosin regulatory light chain 2, skeletal muscle isoform	*MLRS*	2.77	Up	0.042
Myosin regulatory light chain 2, atrial isoform	*MLRA*	2.53	Up	0.011
Laminin subunit alpha-4	*LAMA4*	2.29	Up	0.013
Myosin regulatory light polypeptide 9	*MYL9*	1.77	Up	0.023
Myosin light polypeptide 6	*MYL6*	1.72	Up	0.011
Laminin subunit α-2	*LAMA2*	1.46	Up	0.037
Integrin β-1	*ITB1*	1.37	Up	0.040
Tensin-1	*TENS1*	-1.66	Down	0.018
Ezrin	*EZRI*	-1.68	Down	0.018
Tenascin-X	*TENX*	-1.81	Down	0.012
Collagen alpha-1(IV) chain	*CO4A1*	-2.05	Down	0.005
Filamin-C	*FLNC*	-3.09	Down	0.011
Alpha-actinin-2	*ACTN2*	-3.11	Down	0.041
Laminin subunit α-5	*LAMA5*	-3.22	Down	0.030

Abbreviations: Log2FC, Log 2-Fold Change.

ESM Fig. 1

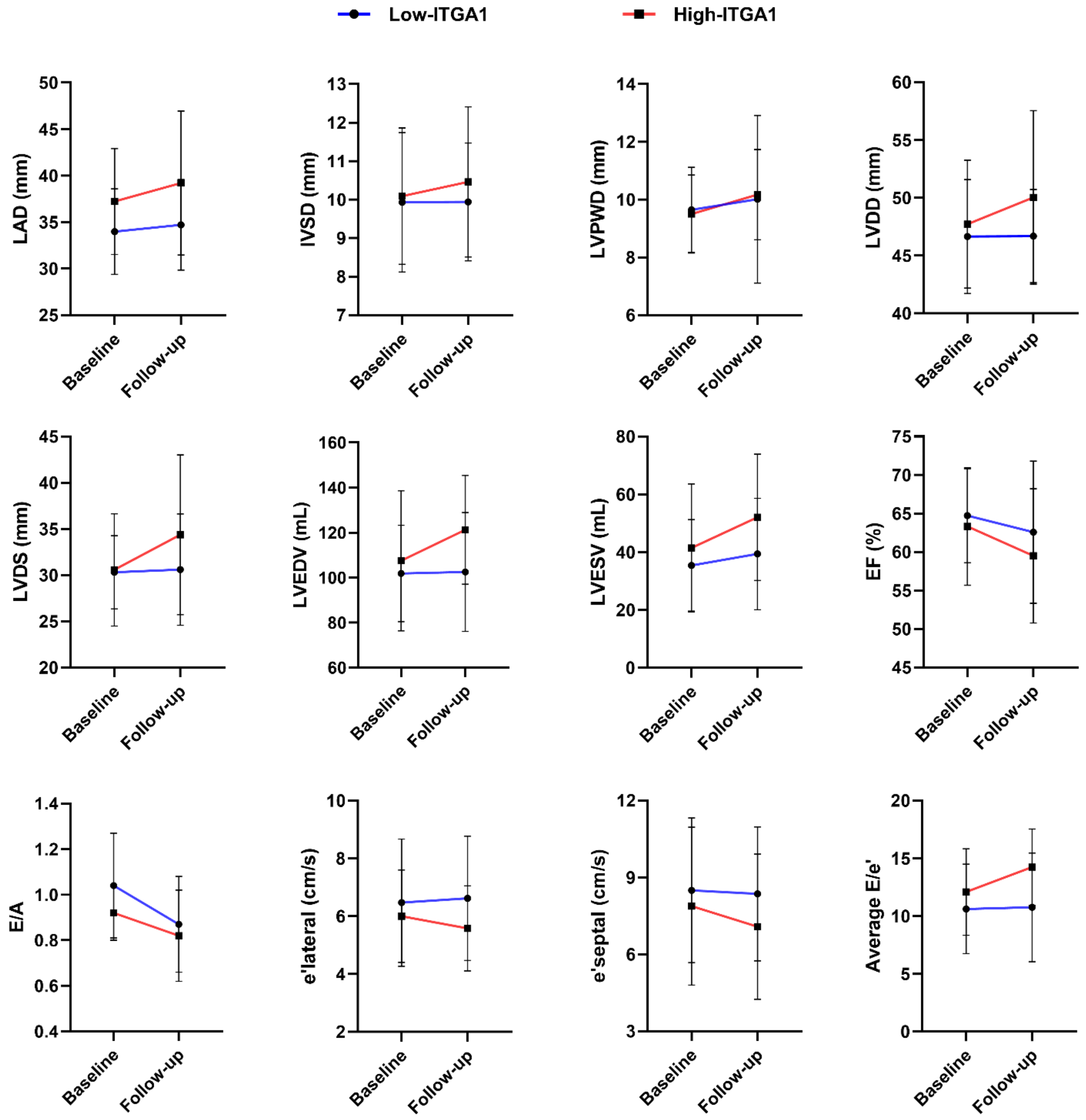

Changes in cardiac structure and function, stratified by plasma ITGA1 levels. Bars represent mean±SD. Abbreviations: A, trans-mitral late diastolic peak velocity; E, trans-mitral early diastolic peak velocity; e′, early diastolic peak velocity of mitral valve at septal or lateral annulus; IVSD, inter-ventricular septal dimension at end-diastole; LVDD, left ventricular end diastolic dimension; LVDS, left ventricular end systolic dimension; LVEDV, left ventricular end diastolic volume; LVESV, left ventricular end systolic volume; LVEF, LV ejection fraction.

ESM Fig. 2

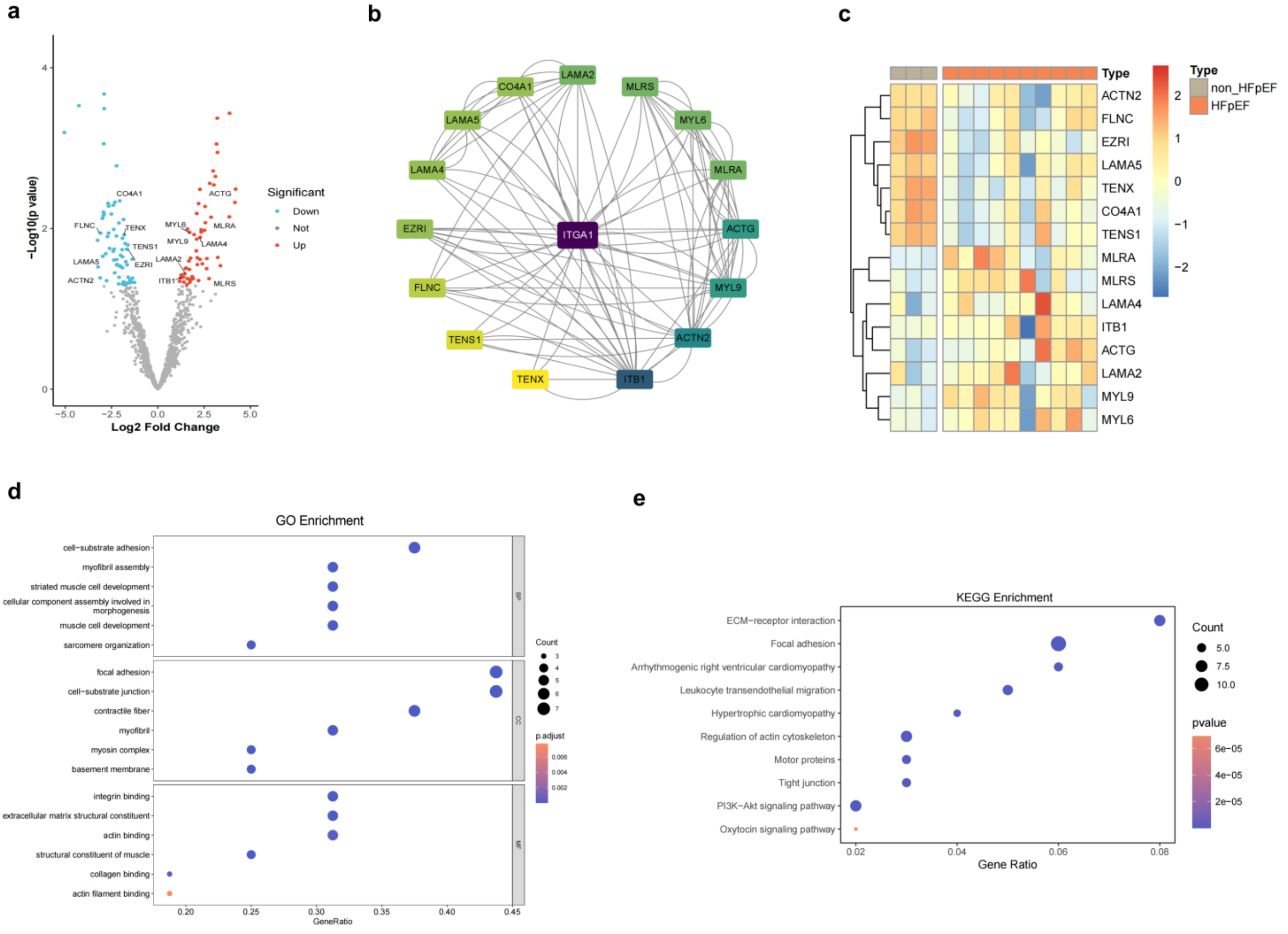

Proteomic analysis of differential protein expression in the left ventricle of T2DM patients with and without HFpEF. **(a)** Volcano plot illustrating the expression of sequenced proteins in T2DM patients with HFpEF (n=10) compared to those without HFpEF (n=3). A total of 124 proteins displayed significant differential expression, with 67 down-regulated (shown in blue) and 57 up-regulated (shown in red). Highlighted genes represent target proteins of ITGA1; **(b)** The network representation illustrates proteins that exhibit strong associations with ITGA1 among the differentially expressed proteins. The strength of the correlation is depicted by the color of the protein names' surrounding boxes, with darker boxes indicating stronger correlations. The complete names of the biomarkers can be found in ESM Table 5; **(c)** The heatmap displays the protein expression levels that exhibit significant correlation with ITGA1 in both patient groups; **(d)** GO analysis of the proteins significantly correlated with ITGA1; **(e)** KEGG analysis of the proteins significantly correlated with ITGA1.
